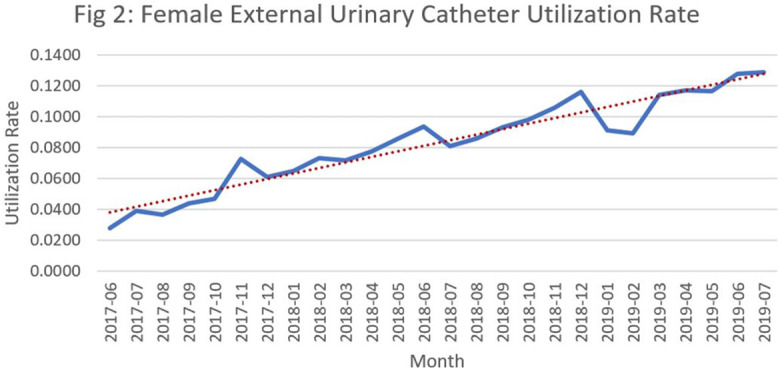# Impact of Female External Urinary Catheter on Indwelling Catheter Use and Catheter-Associated Urinary Tract Infection Rates

**DOI:** 10.1017/ash.2021.11

**Published:** 2021-07-29

**Authors:** Lea Monday, Geehan Suleyman, George Alangaden, Stephanie Schuldt, Catherine Jackman, Christine Halash

## Abstract

**Background:** Catheter-associated urinary tract infections (CED: TIs) are one of the most prevalent healthcare-associated infections. They can lead to bacteremia and increased length of stay, healthcare costs, and mortality. Indwelling urinary catheter (IUC) prevention bundles, nurse-driven removal protocols, and the use of external catheters can help reduce CED: TIs. However, female external urinary catheters (FEUCs) have only recently become widely available. FEUCs were introduced at our institution in July 2017. The purpose of this study was to evaluate the impact of FEUC on IUC utilization ratio and overall CED: TI rate in an 844-bed teaching hospital in southeastern Michigan. **Methods:** We retrospectively evaluated the utilization ratio of FEUCs (female FEUC days per patient days ×1,000) and female IUCs (IUC days per patient days ×1,000), and labia hospital-acquired pressure injury (HAPI) rate due to FEUC from July 2017 through June 2019. We compared the overall (male and female) CED: TI rate per 1,000 IUC days in the preintervention period (January 2016 to June 2017) to the postintervention period (July 2017 to June 2019). **Results:** In total, 4,013 FEUCs were placed during the intervention period. The utilization ratio of FEUC increased by 59% and the utilization ratio of female IUC decreased by 13% over the course of the 2 years. Only 1 HAPI was reported during the observation period at a rate of 0.025% (1 of 4,013). The overall CED: TI rate decreased from 1.60 to 1.40 (*P* = .372). **Conclusion:** Introduction of a FEUC was associated with a decrease in the IUC utilization ratio in female patients with minimal adverse events; however, there was no significant difference in the overall CED: TI rate.

**Funding:** No

**Disclosures:** None

**Figure 1.**
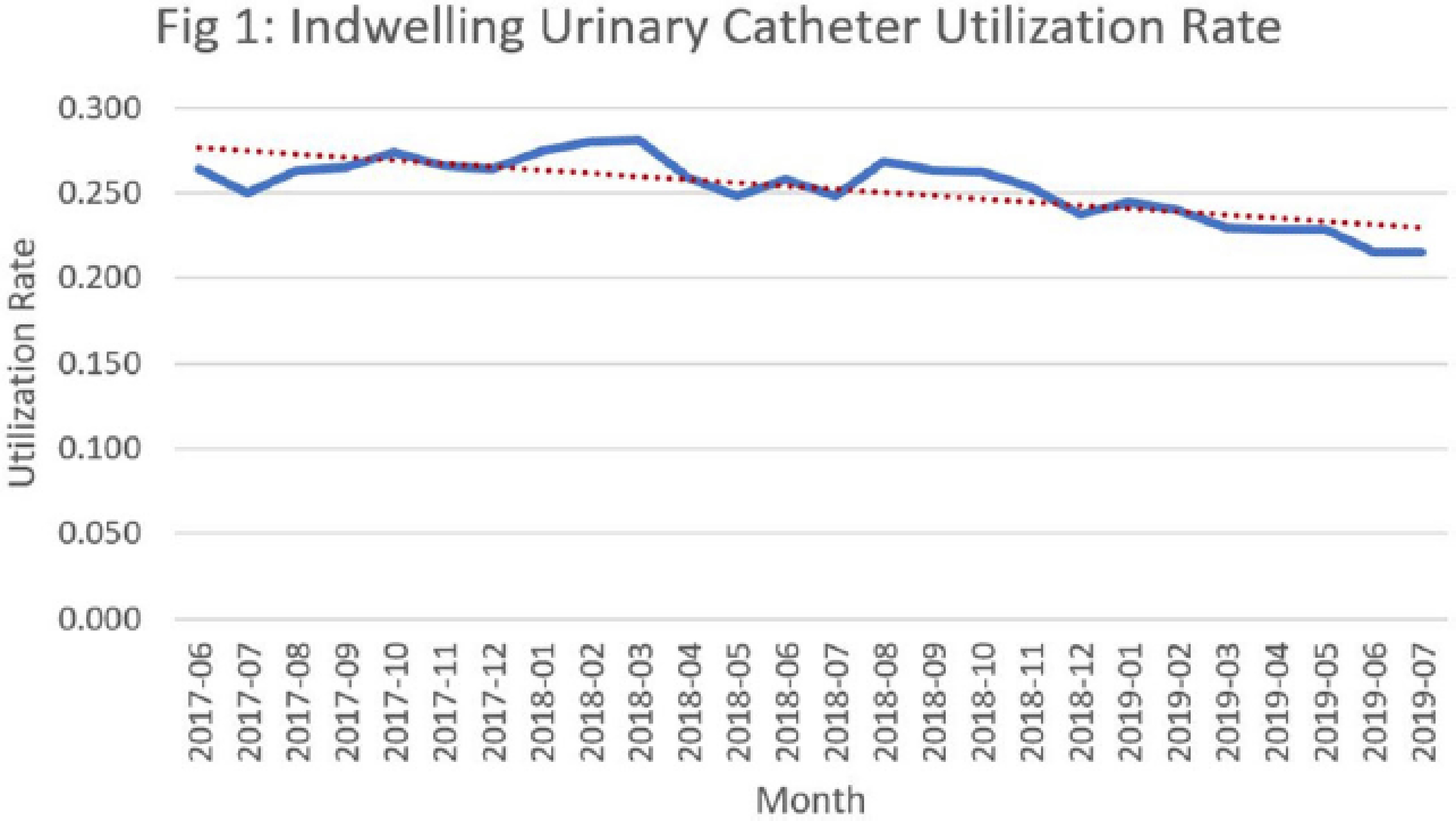

**Figure 2.**